# Tryptophan metabolism circadian regulation: clinical and pharmacological implications

**DOI:** 10.3389/fphar.2026.1943534

**Published:** 2026-10-21

**Authors:** Consuelo Fabi, Sarah Balucchi, Marina Maria Bellet

**Affiliations:** Laboratory of General Pathology, Department of Medicine and Surgery, University of Perugia, Perugia, Italy

**Keywords:** AhR, circadian medicine, clock genes, immune response, indoles, Kynurenines, serotonin

## Abstract

Circadian clocks are endogenous systems that synchronize physiology and behavior to the light-dark cycle, regulating sleep-wake timing, hormonal rhythms, metabolism and immunity. Among metabolic pathways tightly linked to circadian rhythm, tryptophan (Trp) metabolism occupies a central position because its catabolism through the kynurenine (Kyn) pathway, the serotonin (5-HT)-melatonin pathway and the microbiota-derived indole-AhR pathway, produce molecules endowed with significant neuro-modulatory and immunoregulatory activities and directly involved in circadian regulation. Emerging evidence supports a bidirectional relationship between the clock system and Trp metabolism. The molecular clock regulates expression and activity of key Trp enzymes, creating time-of-day variation in Trp flux, Kyn-Trp ratio, serotonin/melatonin synthesis and AhR ligands availability. Conversely, Trp-derived metabolites in turn modulate clock function and downstream physiology, such as diurnal variations in metabolism and immune response. Here we discuss recent studies highlighting how this interplay has important pathophysiological implications and disruption of Trp metabolism oscillatory pattern influences our susceptibility to developing diseases, such as infectious, metabolic and neuropsychiatric diseases. Moreover, we highlight the importance of considering daily fluctuation when using these molecules as biomarkers and interpreting them in a clinical setting. Among circadian-based interventions, such as chronotherapy, light therapy or time-restricted feeding, we propose pharmacological targeting of Trp pathways or pharmacological use of Trp derivatives as a novel approach of circadian medicine, able to improve clinical outcomes across various fields.

## 1 Introduction

Trp is an essential aromatic amino acid whose breakdown produces multiple bioactive molecules exerting many neuro, immune and metabolic functions. In humans more than 95% of dietary Trp is processed through the Kyn pathway, about 1%–2% is converted to 5-HT and melatonin, and a portion is transformed by host and microbial enzymes into indole derivatives (Torrelli-Diljohn et al., 2026). The three main routes of Trp catabolism are controlled by different enzymes and regulatory mechanisms. Therefore, a change in one pathway often alters the availability of Trp for the others and changes downstream signaling. The Kyn pathway starts with an initial rate-limiting step that breaks the indole ring of the Trp converting it in N-formyl kynurenine. This metabolism of Trp is tightly controlled and driven by two distinct heme-containing dioxygenase enzymes: Trp 2,3-dioxygenase (TDO) and indoleamine 2,3-dioxygenases (IDO1 and IDO2). TDO, the principal rate-limiting enzyme that governs systemic Trp catabolism, is constitutively expressed in the liver and is strongly regulated by glucocorticoids and substrate levels (Zhai et al., 2015). IDO1 is induced in immune and epithelial cells by inflammatory signals such as INF-γ, and by innate immune activation, and it contributes to promoting immune tolerance and limiting inflammation (Munn and Mellor, 2013; Ye et al., 2019). IDO2 shares high structural similarity with IDO1 but exhibits markedly lower catalytic activity (Suvieri et al., 2024). Kyn produced by these enzymes is subsequently metabolized into a range of bioactive derivatives, including kynurenic acid (Kyna), quinolinic acid (Quin) and downstream intermediates involved in NAD^+^ biosynthesis. Serotonin (5-HT) pathway is instead governed by the enzyme Trp hydroxylase (TPH), which catalyzes the rate-limiting conversion of Trp to 5-hydroxytryptophan (5-HTP), then decarboxylated to 5-HT. In mammals, there are two distinct *Tph* genes: *Tph1*, which is chiefly expressed in peripheral tissues, especially gut enterochromaffin cells and the pineal gland, whereas *Tph2* is the neuronal isoform expressed in central serotonergic neurons (Walther et al., 2003). A third route converts Trp into indole derivatives through host and microbial enzymes. Commensal bacteria in the colon utilize specific enzymes to break down Trp into several metabolites, which are then absorbed into the bloodstream and exerts immunoregulatory functions in peripheral organs, as well as neurological effects in the central nervous system (Hubbard et al., 2015; Costantini et al., 2020) (Figure 1).

**FIGURE 1:**
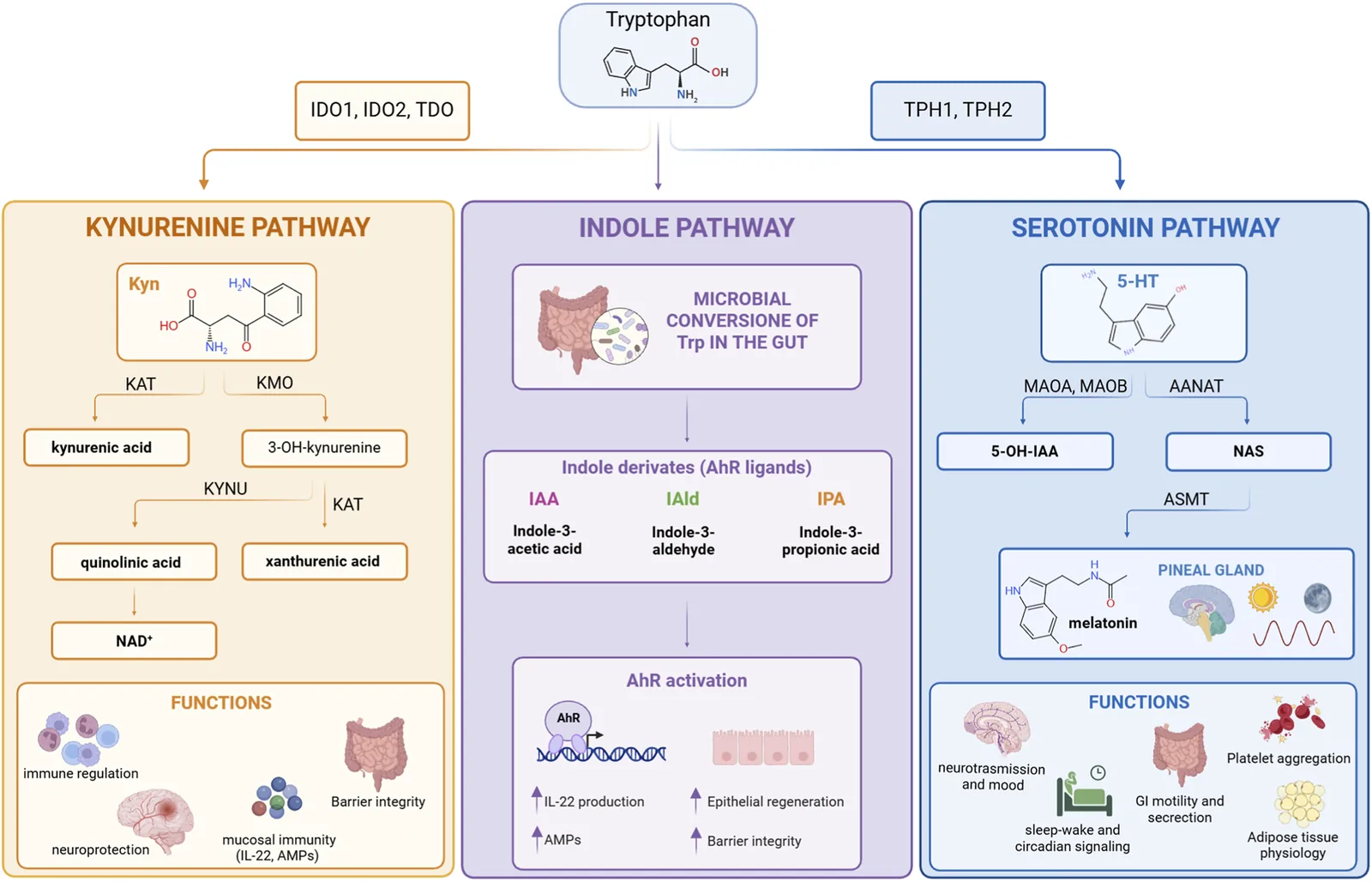
Trp metabolic pathways and their main physiological functions. IDO1, indoleamine 2,3-dioxygenase 1; IDO2, indoleamine 2,3-dioxygenase 2; TDO, Trp 2,3-dioxygenase; TPH1, Tryptophan hydroxylase 1; TPH2, Tryptophan hydroxylase 2; Kyn, Kynurenine; KAT, Kynurenine aminotransferase; KMO, Kynurenine 3-monooxygenase; KYNU, Kynureninase; NAD^+^, Nicotinamide adenine dinucleotide; 5-HT, serotonin; MAOA, Monoamine oxidase A; MAOB, Monoamine oxidase B; 5-OH-IAA, 5-hydroxyindoleacetyc acid; AANAT, serotonin N-acetyl transferase; NAS, N-acetyl-serotonin; ASMT, acetyl serotonin O-methyltransferase. Created in BioRender. Bellet, M. M. (2026) https://BioRender.com/f85z9rm.

In recent years, various studies have highlighted the central role of circadian rhythms in regulating a large part of our metabolic functions (Reinke and Asher, 2019). In the following paragraphs, we aim to describe recent evidence demonstrating the circadian regulation of various branches of Trp metabolism and how this regulation influences the activity of these molecules within the immune system, the central nervous system, and metabolism itself. We will then discuss the diagnostic and therapeutic implications of circadian regulation of Trp metabolism, and how pharmaceutical derivatives of tryptophan can benefit from a circadian medicine approach.

## 2 Circadian rhythms

Circadian rhythms are endogenous biological oscillations characterized by a periodicity of approximately 24 h, synchronized with the environmental light–dark cycle. These rhythms regulate a wide range of physiological processes, including the sleep–wake cycle, thermoregulation, cardiovascular activity, immune function, endocrine secretion, and metabolic homeostasis (Mohawk et al., 2012; Reppert and Weaver, 2002). In mammals, the principal external cue that synchronizes the circadian system is light. Light signals are detected by the master circadian pacemaker, the suprachiasmatic nucleus (SCN), also known as the “master clock,” located in the hypothalamus. From the SCN, timing signals are transmitted to peripheral tissues throughout the body, where an integrated network of tissue-specific endogenous clocks regulates specific biological functions, ensuring a tightly coordinated diurnal physiology across the whole body (Mohawk et al., 2012). Virtually every cell in the body possesses its own circadian machinery, referred to as the molecular circadian clock. The core components of this clock are a group of genes known as clock genes (Cox and Takahashi, 2019). Most clock genes encode proteins and transcription factors that regulate gene expression through interconnected transcriptional-translational feedback loops, generating rhythmic cycles of gene activation and repression over a 24-h period (Bellet and Sassone-Corsi, 2010). The circadian clock plays a crucial role in the development and progression of pathological conditions. Indeed, disruption or misalignment of circadian rhythms, often occurring because of environmental, behavioral, or physiological factors, leads to widespread physiological dysregulation, and has been linked to increased susceptibility to a variety of conditions, including sleep disorders, metabolic dysfunction, cardiovascular disease, immune dysregulation and mood disorders (Allada and Bass, 2021).

Circadian rhythms and Trp metabolism are historically connected since the discovery that Trp serves as the precursor for 5-HT and melatonin, two key molecules involved in regulating the sleep-wake cycle and circadian physiology (Miyamoto et al., 2024). However, their cooperative roles in shaping physiology and circadian rhythms have not been thoroughly investigated. Studies in the last years uncovered how circadian clocks have a role in dictating the oscillatory activity and expression of Trp catabolic enzymes, and the consequent production of bioactive substrates, while Trp-derived metabolites feedback on clock function and modulate circadian homeostasis. These bidirectional links lead to time-of-day variation in immune responses, mood and metabolism (Figure 2).

**FIGURE 2:**
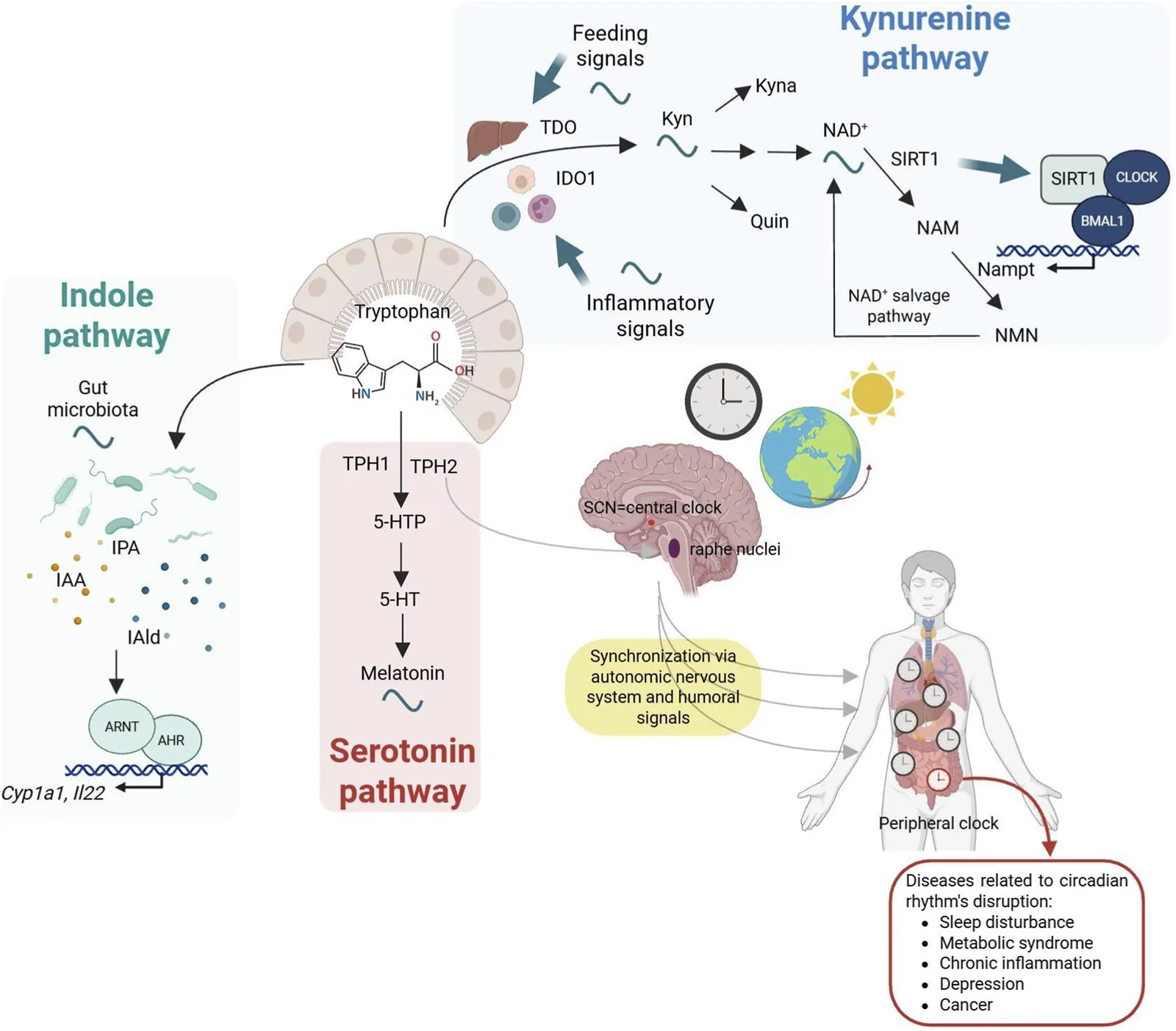
Crosstalk between circadian rhythm and Trp metabolic pathways. Trp, tryptophan; Kyn, kynurenine; Kyna, kynurenic acid; Quin, quinolinic acid; TDO, Trp 2,3-dioxygenase; IDO1, indoleamine 2,3-dioxygenase 1; NAM, nicotinamide; NMN, Nicotinamide mononucleotide; 5-HT, serotonin; 5-HTP, 5-hydroxytryptophan; TPH, Tryptophan hydroxylase; IPA, indole 3 propionic acid; IAA, indole 3 acetic acid; IAld, indole 3 aldehyde; SCN, suprachiasmatic nucleus. Created in BioRender. Bellet, M. M. (2026) https://BioRender.com/zhuo8rj.

### 2.1 Circadian rhythm and the Kyn pathway

Kyn pathway metabolites constitute a group of molecules with distinct biological actions (Badawy, 2017; Cervenka et al., 2017). In the immune system, Kyn and its breakdown products have major immunoregulatory effects. Kyn itself acts as a signaling molecule involved in immune regulation, by activating Aryl hydrocarbon receptor (AhR), a ligand-activated transcription factor that plays central roles in mucosal immunity, barrier maintenance, epithelial repair and xenobiotic metabolism (Stevens et al., 2009). In the central nervous system, Kyn pathway metabolites have opposing effects. Kyna exerts neuroprotective actions through inhibition of excitatory neurotransmission, acting as an antagonist at glutamatergic N-methyl-D-aspartate (NMDA) receptors and as a negative modulator of α7 nicotinic acetylcholine receptors (Schwarcz et al., 2012). Instead, 3-hydroxykynurenine (3-HK) and Quin promotes oxidative stress and act as NMDA agonists with neurotoxic effects (Fathi et al., 2022). Dysregulation of Kyn metabolism, often driven by chronic immune activation and altered TDO/IDO1 activity, results in an imbalance between neuroprotective and neurotoxic metabolites and has been implicated in mood disorders, neurodegenerative diseases, and cognitive dysfunction (Cervenka et al., 2017; Schwarcz and Stone, 2017). The circadian clock regulates the Kyn pathway, causing time of day variation in pathway flux and downstream metabolite balance (Petrus et al., 2022; Moravcova et al., 2022; Gheorghe et al., 2024). Hepatic TDO2, a key determinant of systemic Kyn throughput, displays diurnal changes in mice, entrained by feeding and circadian glucocorticoid release, with peak TDO activity and plasma Kyn during the active phase. Such diurnal regulation confers rhythmicity to Trp metabolites in liver and serum (Morgan et al., 2026). Importantly, such diurnal regulation drives and sustain a robust circadian tone of AhR activity, with important consequences in AhR-dependent physiology (Morgan et al., 2026). Beyond the liver, TDO is also expressed in other tissues, including the placenta (Minatogawa et al., 2003) as well as different brain regions (Haber et al., 1993), where it has been associated to neurodegenerative disorders (Breda et al., 2016; van der Goot et al., 2012; Campesan et al., 2011). In contrast to hepatic TDO, whether TDO expression in these tissues exhibits circadian rhythmicity remains unclear. Likewise, TDO is constitutively expressed in several tumor types, particularly glioblastoma, where its expression contributes to an immunosuppressive tumor phenotype promoting tumor cell survival and invasiveness (Opitz et al., 2011; Pilotte et al., 2012). Extrahepatically, an intrinsic clocks in immune cells (Curtis et al., 2014) can shape IDO1 responsiveness. Indeed, our studies have demonstrated the circadian regulation of IDO1 at the transcriptional level in experimental models. *Ido1* mRNA expression and enzymatic activity follow a diurnal pattern in murine lung and intestine (Stincardini et al., 2023). Such regulation is exerted both in basal conditions, as well as during inflammatory stimuli inducing time-dependent Kyn production and is abrogated in mice with genetic disruption of the circadian core gene *Bmal1* (Stincardini et al., 2023). Macrophages and dendritic cells display circadian variation in cytokine production and signal transduction (Keller et al., 2009; Gibbs et al., 2012), which likely causes the magnitude and timing of IDO1 induction, and therefore local Kyn generation, to differ across the day-night cycle. Under normal conditions, daily rhythms gate cytokine release, leukocyte trafficking and inducible expression of IDO1 (Costantini et al., 2025); when these rhythms are perturbed by shift work, sleep loss or irregular feeding, this temporal control is lost and Trp is more likely to be diverted into the Kyn pathway at inappropriate times (Petrus et al., 2022; Jerigova et al., 2022).

The Kyn pathway is also the major de novo source of NAD^+^, linking Trp metabolism to cellular redox balance, sirtuin activity and mitochondrial function (Schwarcz et al., 2012). Oscillation in Kyn pathway alters cellular NAD^+^ levels and thereby modulates the activity of the NAD^+^ -dependent deacetylase SIRT1. SIRT1 is intimately connected with the circadian system; recruited to promoters of clock-controlled genes in a circadian manner together with the circadian transcription factors CLOCK and BMAL1, SIRT1 modulates circadian gene expression through its histone deacetylase HDAC activity, by repressing transcription in a time-dependent manner (Bellet and Sassone-Corsi, 2010). Moreover, SIRT1 deacetylates core clock components, including BMAL1 and PER2 (Nakahata et al., 2008; Asher et al., 2008), thereby regulating circadian transcriptional amplitude and stability, while its activity is itself driven by oscillations in intracellular NAD^+^ levels generated through the NAMPT-mediated salvage pathway (Nakahata et al., 2009; Ramsey et al., 2009; Bellet et al., 2013).

A substantial proportion of the mechanistic evidence supporting circadian regulation of the Kyn pathway derives from rodent models. Although experiments have also detected circadian regulation of tryptophan metabolism in human cells (Stincardini et al., 2023), direct evidence for tissue-specific rhythmicity of TDO and IDO1 in humans remains limited. The classic human evidence for hepatic TDO rhythmicity is indirect. In 1968 Rapoport and Beisel demonstrated time-of-day-dependent differences in urinary Kyn metabolites following Trp administration, which was interpreted as evidence for rhythmic hepatic TDO activity; however, this study did not directly measure hepatic TDO expression or activity (Rapoport and Beisel, 1968). Human metabolomic studies reported conflicting results with some reports showing rhythmic plasma levels of Trp metabolites (Kervezee et al., 2019; Skene et al., 2018), while no change was observed in other studies (Dallmann et al., 2012). Importantly, circulating metabolites reflect the integrated contribution of liver, immune cells, gut microbiota, kidney and other tissues, so a plasma rhythm should not automatically be interpreted as evidence of hepatic TDO rhythmicity.

Beyond metabolism and immunity, dysregulation of the Kyn pathway has been consistently implicated in neuropsychiatric disorders. Chronic inflammation shifts Kyn metabolism toward the production of neurotoxic metabolites, such as Quin, while reducing the formation of the neuroprotective metabolite kyna, thereby promoting glutamatergic excitotoxicity, oxidative stress, and neuroinflammation (O'Regan et al., 2026). These metabolic alterations have been associated with major depressive disorder, bipolar disorder, schizophrenia, and other mood disorders, which are also characterized by profound circadian disruption (Lovelace et al., 2017; Wang and Serchov, 2026). Collectively, these findings support a bidirectional relationship in which circadian clocks regulate Kyn pathway activity, whereas Kyn metabolism, through its effects on NAD^+^ production, SIRT1 signaling, immune function, and neuroactive metabolite balance, reciprocally contributes to circadian homeostasis. Consistent with this concept, Petrus and colleagues proposed that tryptophan metabolism serves as a physiological integrator of circadian rhythms by coordinating central and peripheral clocks through rhythmic changes in Kyn metabolites and their interactions with metabolic and inflammatory pathways (Petrus et al., 2022).

### 2.2 Circadian rhythm and the 5-HT pathway

5-HT is a molecule with multiple functions, acting as a neurotransmitter in the CNS and as a pleiotropic signaling molecule peripherally, where it regulates gastrointestinal motility, platelet aggregation, systemic energy homeostasis, and adipose tissue physiology (Berger et al., 2009). In the pineal gland 5-HT is enzymatically converted to melatonin, a hormone produced in response to darkness, transducing circadian time cues and modulating sleep-wake cycles’ architecture (Nunzi et al., 2025). For this reason, the 5-HT pathway is tightly interwoven with circadian organization at multiple levels, and timing relationships between 5-HT, melatonin and clock function have important physiological and clinical consequences. Central secretion of 5-HT by the raphe nucleus shows time of day variation in experimental models, thanks to the rhythmic transcriptional regulation of *Tph2* enzyme (Malek et al., 2005; Park et al., 2024). The endogenous clock appears to directly contribute to this rhythmicity. Indeed, a binding site of the circadian protein REV-ERBα on the *Tph2* promoter has been found. Consequently, targeted deletion of *Rev-erbα* in 5-HT neurons was shown to increases 5-HT synthesis and reduces depression-like behavior in mice (Park et al., 2024). In peripheral tissues, initial reports showed circadian expression of *Tph1* in the pineal gland (Sugden, 2003), retina (Liang et al., 2004), testis (Coelho et al., 2019) and gut (Gheorghe et al., 2024). In the pineal gland, melatonin synthesis is a high-amplitude, night-restricted circadian output derived from serotonin (Arendt, 2006). Pinealocytes convert 5-HT to melatonin under sympathetic control from the SCN; the nocturnal rise in melatonin both reflects central clock phase and serves as an endocrine zeitgeber for peripheral tissues, modulating sleep propensity, metabolic processes and immune rhythms (Arendt, 2006). In the gut, *Tph1* expression, at a transcriptional level, is dependent on the presence of microbes (Yano et al., 2015). Thus, microbial influences on enterochromaffin cell function might contribute to daily patterns of peripheral 5-HT production, linking feeding schedules, microbiota activity and host serotonergic signaling.

In humans, several studies demonstrated diurnal variations in peripheral blood serotonin levels (Sauerbier and von Mayersbach, 1976; Malyszko et al., 1994; Pietraszek et al., 1991). Instead, findings regarding circadian variations in platelet 5-HT levels are inconsistent across studies (Wirz-Justice et al., 1977; Modai et al., 1986; Meyerson et al., 1989; Eynard et al., 1993). In the central nervous system, a PET study in healthy men reported diurnal variations in the central serotonergic system, with increased cortical 5-HT1A receptor availability over the course of the day and decreased midbrain 5-HTT availability (Matheson et al., 2015).

Serotonergic signaling also feeds back onto the circadian system. Serotonergic afferents to the SCN, acting through multiple receptor subtypes, modify SCN responsiveness to photic and non-photic cues and can alter phase shifting properties and entrainment dynamics. This modulatory role of 5-HT affects nonphotic entrainment and can change the amplitude or phase of behavioral rhythms under certain conditions, providing a neuronal route for linking mood and circadian timing (Pickard and Rea, 1997). Melatonin receptors in the SCN and in peripheral tissues further mediate feedback effects of the 5-HT-melatonin axis on clock gene expression and circadian physiology (Pfeffer et al., 2022; Cecon et al., 2018).

Circadian disruption perturbs serotonergic biology at several levels. Altered sleep–wake cycles, light exposure at night, shift work and irregular feeding can change TPH expression, 5-HT transporter function and receptor signaling, and they can blunt nocturnal melatonin secretion (Liao et al., 2026; Zisapel, 2018). Importantly, inflammatory activation of the Kyn pathway can divert Trp away from 5-HT synthesis, reducing central and peripheral serotonergic tone. Because immune responsiveness itself is circadian, this shunting may contribute to diurnal patterns of affective and cognitive symptoms in inflammatory and psychiatric disorders (Munn and Mellor, 2013; Lashgari et al., 2023).

### 2.3 Circadian rhythm and the indoles-AhR pathway

Host–microbiota conversion of Trp into indole derivatives is strongly rhythmic, shaped by feeding schedules, intestinal motility, bile secretion and epithelial gene oscillations (Gheorghe et al., 2024; Thaiss et al., 2016). Gut microbial composition and functional gene expression oscillate across the day in experimental models (Thaiss et al., 2014; Leone et al., 2015), so the capacity of the microbiota to produce specific indole ligands, such as indole-3-acetic acid (IAA), indole-3-aldehyde (IAld) and indole-3-propionic acid (IPA), varies with host behavioral cycles and with host clock-regulated intestinal physiology (Thaiss et al., 2016; Liang et al., 2015). These microbial indoles act as ligands or modulators of AhR, whose activation in innate lymphoid cells and epithelial cells promotes IL-22 production and antimicrobial peptide expression, enhances epithelial regeneration and sustains barrier integrity (Zelante et al., 2013; Gutierrez-Vazquez and Quintana, 2018; Kang et al., 2025): such functions depend on availability of microbiota-derived AhR ligands and therefore show diurnal variation (Morgan et al., 2026). In addition, as previously discussed, some Kyn pathway metabolites such as Kyn can themselves activate AhR (Opitz et al., 2011), creating cross-talk between host- and microbe-derived signals. AhR signaling and circadian regulation are structurally and evolutionarily linked. Both AhR and Aryl hydrocarbon Receptor Nuclear Translocator (ARNT), the AhR dimerization partner, are members of the basic helix-loop-helix (bHLH) and PER-ARNT-SIM (PAS) family of transcription factors, to which also belong core clock proteins (Tischkau, 2020; Shimba and Watabe, 2009). AhR activation by exogenous or endogenous ligands, such as Kyn or microbial indoles, alters expression of canonical clock genes and can change the amplitude and phase of peripheral oscillations (Jaeger and Tischkau, 2016; Mukai et al., 2008; Anderson et al., 2013), while disruption of circadian timing in the intestine modifies expression of xenobiotic-metabolizing enzymes, including Cyp1 family members, which are regulated by AhR, and impacts on the luminal environment that shapes microbial metabolism (Deaver et al., 2018; Voigt et al., 2014). This reciprocity means that circadian misalignment associated to irregular feeding, shift work or jet lag can reduce protective indole production, weaken AhR signaling and increase susceptibility to mucosal inflammation. Temporal fluctuations in indole availability and AhR responsiveness can create windows of increased vulnerability to infection or inflammatory exacerbation in disorders such as inflammatory bowel disease, and they influence the timing and efficacy of interventions that target mucosal immunity or microbiota. Though human studies support diurnal variation in microbial composition and microbial-derived metabolites (Thaiss et al., 2014), the extent to which these oscillations causally entrain human tryptophan metabolism or peripheral clocks remains unclear.

Overall, rodent studies have provided important mechanistic evidence demonstrating tissue-specific circadian regulation of tryptophan-metabolizing enzymes and establishing causal links between the circadian clock, tryptophan metabolism, immune responses, and microbial signals. However, these findings cannot necessarily be extrapolated directly to humans. Differences in circadian organization, sleep–wake and feeding patterns, as well as light exposure, metabolic rate, gut physiology, microbiota composition, and experimental conditions may influence the timing and amplitude of tryptophan metabolic rhythms. More broadly, while rodent models are indispensable for mechanistic circadian research, translation to human chronobiology still requires confirmation in appropriately controlled human studies.

## 3 Clinical implications on immune-mediated, metabolic and neurological disorders

The diurnal dynamics of the Kyn pathway, the 5-HT-melatonin production and the indole-AhR axis have clinical relevance and directly impact on immune, metabolic and neuronal homeostasis. Circadian disruption alters immune timing and shifts Trp metabolism in ways that materially affect disease processes. For example, we demonstrated that IDO1 time-dependent induction has relevant effects during infections, as it contributes to modulating immune response against fungal infections, providing direct evidence that the molecular clock temporally controls tryptophan catabolism to optimize host defense mechanisms (Stincardini et al., 2023). Metabolic homeostasis is similarly sensitive to circadian timing of Trp flux. Hepatic metabolism follows feeding-entrained rhythms (Liu et al., 2026; Stokkan et al., 2001; Weger et al., 2026); disruption of feeding schedules or of the hepatic clock can shift systemic Kyn pathway flux and alters NAD^+^ biosynthesis, with downstream effects on SIRT1 activity, mitochondrial function and insulin sensitivity. Experimental models show that circadian misalignment promotes obesity, glucose intolerance and fatty liver (Schrader et al., 2024; Bass and Takahashi, 2010), processes to which altered peripheral 5-HT signaling also contributes (Versteeg et al., 2015). Indeed, gut-derived 5-HT influences insulin secretion, adipogenesis and energy expenditure (Agus et al., 2018), and its daily pattern is entrained by feeding and microbial signals (Yano et al., 2015). Thus, a circadian shift that increases Kyn pathway at the expense of serotonergic or indole/AhR signaling can worsen metabolic outcomes by impairing NAD^+^-dependent metabolic regulation and by altering hormone and adipose tissue biology. However, the best example of how these pathways intersect clinically is found in neuropsychiatric and neurodegenerative disorders. Sleep disturbance and circadian misalignment increase systemic inflammation and Kyn pathway activation; when Kyn pathway flux favors neurotoxic over neuroprotective metabolites, excitotoxicity and oxidative stress rise, which can exacerbate depressive symptoms, cognitive dysfunction and neurodegeneration. Concurrent reduction of central 5-HT availability further links inflammation and circadian disruption to mood and cognitive disorders (Munn and Mellor, 2013; Schwarcz et al., 2012; Platten et al., 2019).

The temporal dimension also matters for diagnosis and biomarker interpretation. Single, untimed measurements of kynurenine, Trp or 5-HT may misrepresent an individual’s pathophysiology because these metabolites and enzyme activities fluctuate with circadian phase and with feeding. If human studies confirm its diurnal oscillation, the Kyn/Trp ratio, a systemic and tissue marker of increased TDO/IDO1 activity widely used in clinic (Badawy and Guillemin, 2019), should be regarded as a time-dependent biomarker, and its interpretation in clinical settings should take temporal variation into account. For example, Kyn/Trp ratio is chronically elevated in inflammatory diseases and correlate with disease severity, immunosuppression and impaired host defense, reflecting both sustained immune activation and dysregulated timing of enzyme induction (Munn and Mellor, 2013; Platten et al., 2019). In oncology, tumor-associated TDO/IDO1 activity and resulting Kyn production promote local immune tolerance and can reduce responsiveness to immunotherapy (Liu et al., 2023; Chen et al., 2022; Karayama et al., 2021); because Kyn pathway enzymes and immune checkpoints are themselves under temporal control, the efficacy of immune therapies may depend on circadian context. Therefore, longitudinal sampling may improve its reliability as biomarkers in clinical research and may refine patient selection and timing for therapies targeting Trp metabolism.

## 4 Pharmacological approaches of circadian medicine

Given the central involvement of the circadian system in the regulation of physiological homeostasis and considering that circadian dysregulation is associated with numerous pathological conditions, increasing attention has been directed toward the identification of therapeutic strategies aimed at restoring circadian rhythmicity (Allada and Bass, 2021; Peeples, 2018). In this context, modulation of the circadian system represents a promising and innovative therapeutic approach for the treatment of many diseases (Ruan et al., 2021; Costantini et al., 2020; Miller and Hirota, 2020). Currently, circadian medicine encompasses three main approaches. The first is chronotherapy, also called “clocking the drug” approach, which aims to optimize the timing of drug administration according to circadian rhythms in order to maximize therapeutic efficacy while minimizing adverse effects. The second approach defined “training the clock”, involves non-pharmacological modulation of circadian rhythms through lifestyle interventions, including regulation of sleep–wake schedules, dietary timing, physical activity, and controlled exposure to natural or artificial light. These environmental and behavioral interventions can contribute to the resynchronization of disrupted circadian oscillators and improve systemic physiological function. The third strategy focuses on the pharmacological targeting of the molecular circadian clock or “drugging the clock” approach. Numerous genetic, biochemical, and pharmacological studies have identified several bioactive compounds, commonly referred to as “small molecules,” capable of modulating circadian clock function. These molecules act on specific components of the molecular clock and have emerged as potential therapeutic candidates to treat disease where there is a disruption of circadian clock.

From a translational perspective, the tight coupling between Trp metabolic pathways and circadian timing supports chronotherapeutic approaches to Trp derivatives studied for potential pharmacological applications (Platten et al., 2019; Modoux et al., 2021). Indeed, the mechanistic links described between the two pathways create opportunities for circadian-based interventions. Hypothetically, chronotherapeutic approaches applied to modulators of Trp metabolism, as well as nonpharmacological entrainment, such as light therapy or time-restricted feeding approaches, could restore beneficial timing of Trp fluxes and potentiate pharmacological interventions by re-establishing favorable windows for immune tolerance versus activation, metabolic regulation and mucosal defense. Similarly, interventions that restore host-microbiota circadian alignment, like feeding schedules, manipulation of microbial communities towards indole-producing taxa, or timed administration of selective AhR ligands, could be applied to reinforce mucosal defenses and normalize circadian immune rhythms (Bautista and Lopez-Cortes, 2025).

However, despite evidence highlighting the need to consider timing for clinical benefit, significant challenges remain. Interindividual variability in circadian profile, linked to biological sex, age, chronotype and sleep–wake behavior, and in the composition of the gut microbiota, associated to diet habits and meal timing, may influence the phase and amplitude of metabolic rhythms and therefore modify the response to time-dependent interventions (Weger et al., 2019; Weger and Gachon, 2019; Pais et al., 2023). Therefore, personalized interventions chronotype-driven, associated with rigorously designed clinical trials are necessary to drive optimization of therapeutic timing. For example, although TDO/IDO1 represent attractive targets and small molecules inhibitors targeting these enzymes hold promises in diverse diseases such as neurodegeneration, autoimmunity and cancer (Platten et al., 2019), there are currently no clinical studies demonstrating that inhibition at a particular circadian phase improves therapeutic efficacy. Similarly, microbiome-directed interventions are an emerging area with multiple therapeutic opportunities (Sorbara and Pamer, 2022; Nagayama et al., 2025; Gilbert et al., 2025). Dietary modification, prebiotics, probiotics, or other microbiota-targeted approaches could potentially alter microbial tryptophan metabolism and thereby influence host kynurenine and indole pathways. Although experimental studies provide evidence for bidirectional interactions between the microbiota and circadian physiology (Teichman et al., 2020), whether manipulation of the microbiota can reproducibly modify human circadian tryptophan metabolism or improve treatment outcomes remains unknown.

## 5 Conclusion

The reciprocal regulation between circadian clocks and Trp metabolism integrates temporal control of immune function, brain signaling, and metabolism over the day. Preclinical studies suggest that disruption of circadian timing could shifts Trp metabolism toward harmful pathways, including increased Kyn production, imbalances in NAD^+^, reduced serotonin and melatonin synthesis, and altered indole-AhR signaling. These changes may contribute to immune, metabolic, and neuropsychiatric disorders. Considering the time of day when measuring Trp metabolites could improve the interpretation of biomarkers and guide more effective treatments. Strategies such as lifestyle-based circadian alignment, timed drug administration, microbiome-targeted approaches, and therapies that target circadian clocks or Trp pathways could help restore healthy Trp metabolism and improve clinical outcomes. Future studies should identify the best times for sampling and treatment, clarify how these mechanisms differ across tissues and cell types, and evaluate circadian-based therapies in clinical settings.
